# Genome Sequence of the Fructan-Degrading Organism *Marinimicrobium* sp. Strain LS-A18, Isolated from a Marine Solar Saltern

**DOI:** 10.1128/genomeA.00776-13

**Published:** 2013-10-03

**Authors:** Wei-Dong Lu, Hai-Qiang Pang, Li-Zhong Guo

**Affiliations:** Shandong Provincial Key Laboratory of Applied Mycology, College of Life Sciences, Qingdao Agricultural University, Qingdao, People’s Republic of China

## Abstract

*Marinimicrobium* sp. strain LS-A18 is a fructan-degrading organism isolated from a brine sample from a marine solar saltern in Jiaozhou Bay, China. The draft genome sequence of this bacterium is 3,815,107 bp in length, with a G+C content of 59.03%. To our knowledge, this is the first genome announcement of a fructan-degrading strain of the genus *Marinimicrobium*.

## GENOME ANNOUNCEMENT

As fossil fuels on the earth are being depleted, much effort has been devoted to screening the carbohydrate polymer-degrading microorganisms that can efficiently convert plant biomass into biofuels. Besides cellulose and hemicellulose, fructan is another promising candidate for the production of biofuels. Fructans are linear and branched polymers of fructose attached to a sucrose core, and 15% of all flowering plant species, including Jerusalem artichoke, chicory, and dahlia, store fructans as the carbohydrate reserve (1).

Lim et al. (2) first described the genus *Marinimicrobium* based on two novel species in this genus. So far, *M. agarilyticum*, *M. koreense*, *M. locisalis* (3), and *M. haloxylanilyticum* (4) are the four described species in the genus. *Marinimicrobium* sp. strain LS-A18, an inulin-degrading bacterium, was isolated from marine solar saltern in Jiaozhou Bay, China (5). It can grow on a number of carbohydrates and carbohydrate polymers, such as inulin, levan, and carboxymethyl cellulose, and high yields of extracellular inulinase and carboxymethyl cellulase can be detected in the supernatant of culture broth (5, 6). The biochemical properties of these hydrolytic enzymes are unique and different from those produced by terrestrial microorganisms. To gain insight into the mechanisms involved in fructan degradation and evaluate the potential for hydrolytic enzyme production, a draft genome sequence of *Marinimicrobium* sp. strain LS-A18 is presented here.

The draft genome sequence of *Marinimicrobium* sp. LS-A18 was generated using an Illumina HiSeq 2000 with a whole-genome shotgun (WGS) strategy. A short-insert paired-end (PE) library with an average insert size of 300 bp and a long-insert mate-pair (MP) library with average insert size of 3 kb were constructed. The Illumina PE library generated 10,440,850 reads totaling 1,054.5 Mb with an average genome coverage of 276.4×, while the Illumina MP library generated 11,625,984 reads totaling 1,174.2 Mb with an average coverage of 307.8×. All reads were assembled into 14 large contigs with an N_50_ contig length of 1,095,460 bp and an N_90_ contig length of 236,722 bp. The draft genome assembly was 3,815,107 bp in length, with a mean G+C content of 59.03%.

The prediction of open reading frames (ORFs), tRNAs, and rRNAs was performed by using Glimmer 3.02, tRNAscan-SE 1.3, and RNAmmer 1.2 (7–9), respectively. Genome annotation was carried out with the RAST server (10). A total of 3,194 ORFs, 46 tRNAs, and 3 rRNAs were predicted. Among these, 1,645 coding sequences (CDSs) belong to 412 RAST subsystems, while 1,493 CDSs did not belong to any subsystems. Of those in RAST subsystems, 1,534 CDSs correspond to nonhypothetical proteins.

The genome contains numerous genes encoding homologs of enzymes that deconstruct plant biomass. Genes encoding putative fructan-degrading enzymes, such as β-d-fructofuranosidase, inulinase, and levanase, as well as some xylan- and cellulose-degrading enzymes, were identified and are currently being analyzed. To our knowledge, this is the first genome announcement for the genus *Marinimicrobium*, which will provide a template for further phylogenetic and comparative genomic studies of this genus.

### Nucleotide sequence accession number.

This whole-genome shotgun project has been deposited at GenBank under the accession no. AWEP00000000.
